# Seawater temperature and buffering capacity modulate coral calcifying pH

**DOI:** 10.1038/s41598-018-36817-y

**Published:** 2019-02-04

**Authors:** Weifu Guo

**Affiliations:** Department of Geology and Geophysics, Woods Hole Oceanographic Institution, Woods Hole, MA 02543 USA

## Abstract

Scleractinian corals promote the precipitation of their carbonate skeleton by elevating the pH and dissolved inorganic carbon (DIC) concentration of their calcifying fluid above that of seawater. The fact corals actively regulate their calcifying fluid chemistry implies the potential for acclimation to ocean acidification. However, the extent to which corals can adjust their regulation mechanism in the face of decreasing ocean pH has not been rigorously tested. Here I present a numerical model simulating pH and DIC up-regulation by corals, and use it to determine the relative importance of physiological regulation versus seawater conditions in controlling coral calcifying fluid chemistry. I show that external seawater temperature and buffering capacity exert the first-order control on the extent of pH elevation in the calcifying fluid and explain most of the observed inter- and intra-species variability. Conversely, physiological regulation, represented by the interplay between enzymatic proton pumping, carbon influx and the exchange of calcifying fluid with external seawater, contributes to some variability but remain relatively constant as seawater conditions change. The model quantitatively reproduces variations of calcifying fluid pH in natural *Porites* colonies, and predicts an average 0.16 unit decrease in *Porites* calcifying fluid pH, i.e., ~43% increase in H^+^ concentration, by the end of this century as a combined result of projected ocean warming and acidification, highlighting the susceptibility of coral calcification to future changes in ocean conditions. In addition, my findings support the development of coral-based seawater pH proxies, but suggest the influences of physicochemical and biological factors other than seawater pH must be considered.

## Introduction

Ocean acidification is considered a serious threat to the health of coral reef ecosystems, because it reduces the concentration of carbonate ions ([CO_3_^2−^]) that corals need to build their calcium carbonate (aragonite) skeleton. It is estimated that seawater pH has already decreased by about 0.1 unit since the preindustrial era and could decrease by another 0.14–0.43 units by 2100^1–3^. This corresponds to about 22–56% reduction of seawater [CO_3_^2−^], and implies ~48% decreases in aragonite precipitation rates based on the kinetic parameters determined in abiotic experiments^4^. Nevertheless, while the negative impact of ocean acidification on coral calcification is generally accepted, projecting the exact magnitude of this impact is challenging. Laboratory manipulation experiments and field measurements show large variations in calcification responses among different coral species and reef systems, ranging from decreasing dramatically with decreasing seawater aragonite saturation state (Ω_arag_), to not changing significantly with Ω_arag_, to increasing under moderately decreased Ω_arag_^5,6^. Such variability arises in part from the fact that corals do not precipitate their carbonate skeleton directly from ambient seawater but within an extracellular calcifying fluid whose chemistry is strongly regulated by corals^7–10^. This calcifying fluid, located between the coral skeleton and its calicoblastic cell membrane, constitutes an internal calcification environment significantly different from the ambient seawater, making it difficult to extrapolate changes in seawater chemistry to those in coral calcifying fluid.

**Figure 1:**
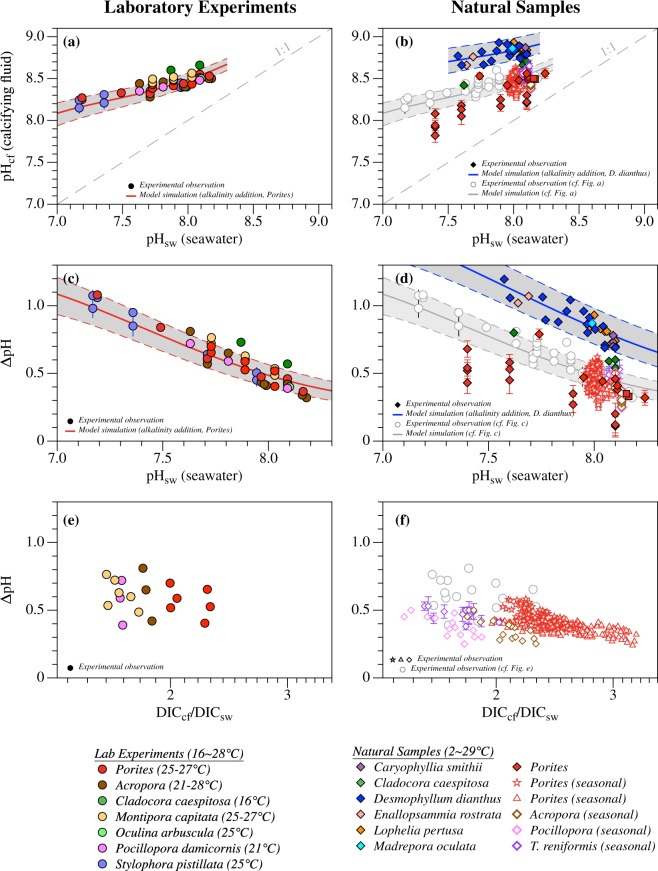
Representative experimental constraints on the calcifying fluid pH and DIC concentrations among different coral species^11–24,27,28^. (**a**–**d**) Despite the large inter- and intra-species variations, both laboratory experiments rearing corals under high pCO_2_ conditions and measurements of some natural coral samples show consistent negative correlations between calcifying fluid pH elevation (ΔpH) and pH of the seawater (pH_sw_) from which corals grow. Such correlations are consistent with predictions from alkalinity addition calculations (colored lines and shaded area), supporting seawater buffering capacity exerts fundamental control on pH elevation in coral calcifying fluid. (**e**,**f**) Coral calcifying fluid DIC concentrations as constrained by geochemical proxies are also elevated relative to the seawater. The extents of DIC elevation (DIC_cf_/DIC_sw_) show weak correlations with the pH elevations (ΔpH) in laboratory cultured corals, but the two appear to exhibit strong negative correlations in their seasonal variations in natural specimens. During the alkalinity addition calculations, constant amounts of alkalinity (450 and 700 μmol·kg^−1^ for *Porites* and *D*. *dianthus*, respectively) were added to seawaters of the same temperatures and DIC concentrations but different pH and thus different buffering capacities (T = 26 °C and 8.3 °C, DIC = 2024 and 2212 μmol kg^−1^ for *Porites* and *D*. *dianthus*, respectively, see text). The shaded areas show the range of predictions with different amounts of alkalinity added (400–550 and 550–900 μmol kg^−1^ for *Porites* and *D*. *dianthus*, respectively).

Most notably, geochemical proxy data suggest both pH (pH_cf_) and dissolved inorganic carbon (DIC_cf_) concentration of coral calcifying fluid are elevated relative to seawater, e.g., by up to 1.2 pH units^11–31^ and a factor of 3.2^11,12,16–18,27–32^, respectively (Fig. 1). In comparison, *in vivo* measurements with pH/CO_3_^2−^ microelectrodes and pH-sensitive dyes show elevated pH_cf_^6,14,33–37^ but DIC_cf_ concentrations similar to seawater values in some corals^36^, suggesting more dynamic carbonate chemistry in coral calcifying fluid^37^. The up-regulation of pH and DIC, achieved in part via enzymatic H^+^ transport and CO_2_ diffusion across the calicoblastic cell membrane, increases CO_3_^2−^ concentration in coral calcifying fluid and thus leads to higher rates of calcification relative to that occurring in the external seawater. The ability of corals to actively regulate their calcifying fluid chemistry has raised questions about the potential for coral acclimation to ocean acidification^13,16–18,23,29,30,38^, confounding efforts to project coral calcification response to changing seawater conditions and the trajectory of coral reef ecosystems in the 21st century.

Variations in the extents of pH and DIC up-regulation among different coral species and colonies further hinder quantitative projections of the impact of ocean acidification. Although laboratory manipulation experiments rearing corals under high pCO_2_ conditions (e.g., *Porites*, *Acropora*, *Stylophora pistillata*) show negative correlations between the extents of coral calcifying fluid pH elevation (i.e., ΔpH = pH_cf_–pH_sw_) and seawater pH^11,14,18,19,21,22,24^ (as first pointed out by ref.^19^), such correlations are not consistently present in natural coral specimens (Fig. 1). Only natural cold-water corals (e.g., *Desmophyllum dianthus*, *Lophelia pertusa*) exhibit a similar trend that is offset to higher ΔpH values^15,20,25^. In contrast, studies of natural reef-building corals (e.g., *Porites*) suggest factors other than seawater pH also affect coral calcifying fluid pH. For example, both *in situ* mesocosm experiments and examination of natural coral colonies at CO_2_ seep sites show *Porites* corals can maintain their calcifying fluid pH (i.e., pH homeostasis) irrespective of moderate decreases in seawater pH, e.g., down to ~7.7^13,23^. Meanwhile, *Porites* corals in some tropical reefs show large seasonal variations in their calcifying fluid pH over relatively limited changes in seawater pH (e.g., at Davies Reef, Ningaloo Reef, and Havannah Island)^12,16^. These seasonal variations, also observed in natural *Acropora yongei*, *Pocillopora damicornis* and *Turbinaria reniformis* colonies^17,28^, correlate with changes in seawater temperature (i.e., lower pH_cf_ at higher temperatures) and were interpreted to reflect corals’ active modulation of their calcifying fluid chemistry in responses to temperature^12,16,17,28^. In addition, corals cultured at the same seawater pH but different DIC concentrations (e.g., *Stylophora pistillata*, *Acropora yongei*, *Pocillopora damicornis*) show large variations in their calcifying fluid pH, supporting seawater pH is not the sole control of coral calcifying fluid pH^30,33^.

Similar variations are also observed in coral calcifying fluid DIC elevation. Geochemical proxy data, particularly the combined boron isotope and B/Ca analysis of coral skeletons, suggest that DIC concentrations in coral calcifying fluid range from 1.4 to 3.2 times of seawater values among different species, e.g., *Porties*, *Acropora yongei*, *Goniopora sp*., *Pocillopora damicornis*, *Stylophora pistillata*, *Turbinaria reniformis*^11,12,16–18,27–31^, with seasonal variations that negatively correlate with pH_cf_^12,16,17,28^. However, *in vivo* microelectrode measurements imply DIC concentrations in the calcifying fluid are similar to that of the ambient seawater in some species, e.g., *Acropora millepora*, *Orbicella faveolata*, *Turbinaria reniformis*^36^.

To date, our understanding of the exact controls on coral calcifying fluid pH and DIC concentration is very limited. The variations observed among different species have been broadly attributed to species-specific physiological effects. Here I present a numerical model simulating the regulation of calcifying fluid chemistry by corals, and determine the relative importance of physiological regulation and seawater conditions in controlling the calcifying fluid chemistry.

## Results

### A physicochemical model of coral calcifying fluid chemistry regulation

At given temperature and salinity, pH changes in aqueous solutions are controlled by two factors: (1) the solution buffering capacity, which quantifies its resistance to pH change and, for seawaters, is dominated by the buffering capacity of the DIC system; (2) the specific processes involved in the modulation of solution chemistry. Accordingly, pH elevation in coral calcifying fluid is expected to be directly related to the buffering capacity of its DIC system as alluded in previous studies^36,39^, and can be calculated based on the corresponding changes in coral calcifying fluid pH when fluid alkalinity (TA) and DIC are altered by different physical and biological processes^40,41^:

1 $${\rm{\Delta }}pH=(\frac{\partial pH}{\partial TA})\times {\rm{\Delta }}TA+(\frac{\partial pH}{\partial DIC})\times {\rm{\Delta }}DIC$$

where the slopes $(\frac{\partial pH}{\partial TA})$ and $(\frac{\partial pH}{\partial DIC})$ denote the sensitivities of the calcifying fluid pH to changes in fluid TA and DIC and are related to the commonly defined buffering factors β_TA_ and β_DIC_^40,42^:

2 $${\beta }_{TA}=-\,{(\mathrm{ln}10\times \frac{\partial pH}{\partial TA})}^{-1},\,\,{\beta }_{DIC}=-\,{(\mathrm{ln}10\times \frac{\partial pH}{\partial DIC})}^{-1}$$

Because β_TA_ and β_DIC_ are similar in magnitude under typical seawater conditions (i.e., β_TA_ ≅ −β_DIC_), pH elevation in coral calcifying fluid can be approximated as:

3 $${\rm{\Delta }}pH\cong -\,0.434\times \frac{1}{{\beta }_{TA}}\times ({\rm{\Delta }}TA-{\rm{\Delta }}DIC)$$

Therefore, at a given temperature, the elevation of coral calcifying fluid pH is expected to negatively correlate with 1/β_TA_. This prediction is consistent with the trends observed in the experimental data. Regardless of the species they belong to, corals that grow in seawaters of similar temperature and buffering capacity show similar extents of pH elevation (Fig. S1).

Alkalinity addition calculations which simulate the enzymatic pumping of H^+^ out of coral calcifying fluid also supports this interpretation (Fig. 1). In these calculations conducted at the average growth conditions of *Porties* and *D*. *dianthus*, hypothetical seawaters of the same temperature, salinity and DIC concentration but of different pH and thus different buffering capacities were generated. Addition of constant amounts of alkalinity to these seawaters quantitatively reproduces the negative ΔpH~pH_sw_ correlations observed within each species (Fig. 1a–d). The variations of ΔpH at given pH_sw_ within each species can also be reproduced by varying the amount of alkalinity added, e.g., by −21~28%.

**Figure 2:**
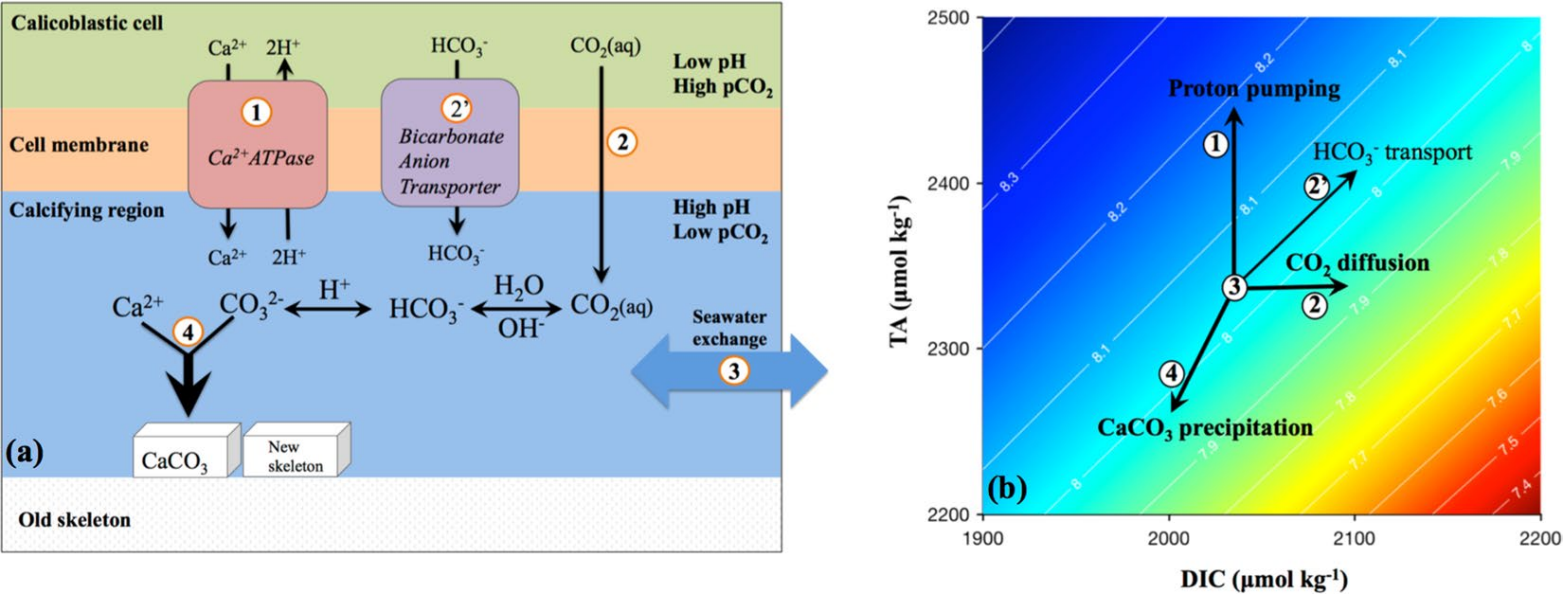
Key processes involved in coral calcification and their effects on the calcifying fluid chemistry. (**a**) Schematics of coral calcification^43^. Numbered are the four key processes involved in coral calcification that were explicitly simulated in this study: (1) proton pumping by Ca-ATPase; (2) diffusion of CO_2_ across the calicoblastic cell membrane induced by the pH and CO_2_ gradients; (3) exchange of the calcifying fluid with external seawater; (4) aragonite precipitation from the calcifying fluid. Also shown is the bicarbonate transport via Cl^−^/bicarbonate anion transporter which has been proposed as another potential source of carbon for coral calcification^7,32,52^. (**b**) Each of these processes modulates TA and/or DIC concentration of coral calcifying fluid differently, leading to corresponding changes in the calcifying fluid pH^43,49,51^ (contour lines, see text). As an illustration, carbonate chemistry calculation in (b) was performed at 25 °C and with a salinity of 35. Figures are modified after refs^43,49^.

The above alkalinity addition calculation is, however, an oversimplification of coral calcification, and does not include many other processes that can affect calcifying fluid chemistry or consider the effects of temperature change. To account for these factors, I construct a numerical model of coral calcification. This model builds on previous theoretical studies^6,15,35,39,43–49^ and simulates four key processes that are directly involved in coral calcification^43^ (Fig. 2a): (1) enzymatic proton pumping, e.g., by Ca^2+^ATPase which pumps Ca^2+^ across the calicoblastic cell membrane removing two H^+^ for every Ca^2+^ transported into the calcifying fluid^50^; (2) diffusion of CO_2_ across the calicoblastic cell membrane induced by the pH and CO_2_ gradients between the cell and the calcifying fluid; (3) exchange between the calcifying fluid and external seawater; (4) aragonite precipitation from the calcifying fluid.

Each of these processes modulates the TA and/or DIC concentrations of coral calcifying fluid differently, leading to corresponding changes in calcifying fluid pH^43,49,51^ (Fig. 2b). For example, enzymatic proton pumping increases [TA]_cf_ without affecting [DIC]_cf_, and is the main driver for the elevation of calcifying fluid pH. In contrast, diffusion of CO_2_ only increases [DIC]_cf_, while aragonite precipitation decreases both [TA]_cf_ and [DIC]_cf_ with a 2:1 molar ratio. Both of these latter processes decrease calcifying fluid pH. At the same time, the exchange of coral calcifying fluid with external seawater brings the calcifying fluid TA and DIC closer to seawater values and mitigates any changes in coral calcifying fluid chemistry induced by other processes. Among these processes, the rate of aragonite precipitation (R_cf_) is expected to vary as a function of aragonite saturation state in the calcifying fluid (Ω_cf_) and temperature:

4 $${{\rm{R}}}_{{\rm{cf}}}={\rm{k}}\times {({{\rm{\Omega }}}_{{\rm{cf}}}-1)}^{{\rm{n}}}$$

where k and n are the temperature-dependent rate constant and reaction order for aragonite precipitation^4^. Note, besides CO_2_ diffusion, transport of HCO_3_^−^ into the calcifying fluid via Cl^−^/bicarbonate anion transporter has also been proposed as a potential source of carbon for coral calcification^7,32,52^. Model simulations including this carbon flux yield similar results and are presented in the SI (Fig. S2).

**Figure 3:**
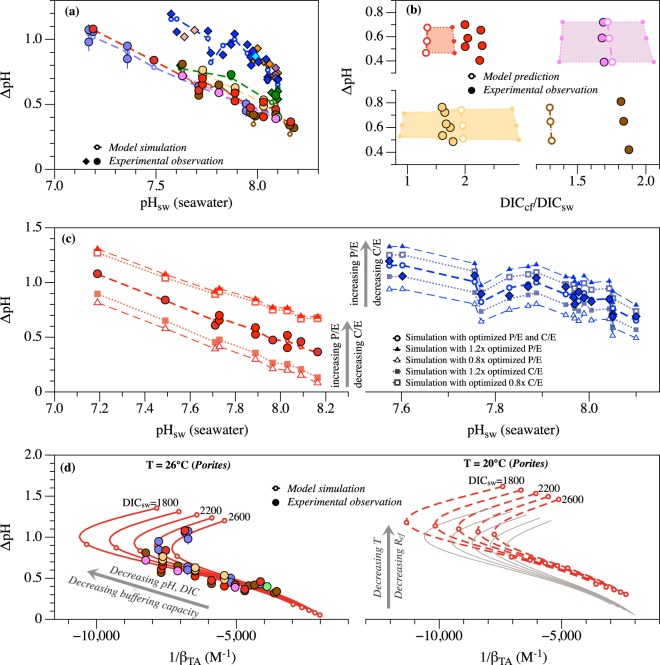
Model simulation of the regulation of calcifying fluid chemistry by corals. (**a**) Comparison between the model simulated calcifying fluid pH elevation (open circles) and the experimentally measured values for each species^11,14,15,18–22,24^ (filled symbols). The open circles represent the best-optimized model results and are connected by dashed lines for better illustration. (**b**) Comparison between the model predicted calcifying fluid DIC elevation and the experimentally measured values on the same corals^11,18^. The large open circles denote the predictions from the best-optimized model parameters, and the shaded areas denote the range of predictions from ‘less optimal’ model parameters whose predicted pH_cf_ fall within two standard error (i.e., 0.02 pH unit) of the predictions from the best-optimized parameters. (**c**) Control of physiological regulation (i.e., P/E, C/E ratios) on the pH elevation in coral calcifying fluid, illustrated with *Porites* and *D*. *dianthus* as examples. Higher P/E and lower C/E ratios are predicted to lead to higher pH elevation. (**d**) Control of seawater physicochemical conditions (e.g., pH, DIC, T) on the pH elevation in coral calcifying fluid, illustrated with *Porites* as an example. Lower seawater pH and DIC lead to lower buffering capacity and thus higher pH elevation in coral calcifying fluid. Lower temperatures are also predicted to lead to higher pH elevation mainly due to slower rates of aragonite precipitation in the calcifying fluid. The model simulations in (**d**) were conducted with the best-optimized parameters for *Porites*, with seawater pH and DIC concentrations varying from 7 to 8.5, and 1800 to 2600 μmol·kg^−1^ respectively. The open circles on the model lines in (**d**) represent the results when pH_sw_ = 7, 7.5, 8 and 8.5, respectively. For comparison, the model predictions at 26 °C (grey lines) are shown next to the predictions at 20 °C (dashed line) in the right panel of (**d**).

Three key parameters are involved in this model, corresponding to the respective fluxes of proton pumping (P), CO_2_ diffusion (C) and the exchange of calcifying fluid with external seawater (E). There are no direct experimental constraints on any of these parameters. I estimate these parameters by assuming they are the same for all coral individuals within the same species and optimizing their values to reproduce the ΔpH~pH_sw_ relationship observed for each species (Fig. 3, Methods). The best estimated P, C and E values, representing the average values of each species, range from 0.35 to 7.7 μmol·m^−2^·s^−1^, 0.17 to 4.6 μmol·m^−2^·s^−1^ and 0.015 to 3.1 g·m^−2^·s^−1^, respectively, for different coral species (Table S1). With the dimension of the calcifying space assumed in the model (i.e., 3 μm thick, Methods), the estimated exchange fluxes correspond to calcifying fluid turnover time of 1 s to 3.4 min, which lies on the low end of existing constraints (i.e., <2 min^52^ to <5.7 h^47^). Note, however, besides these best-optimized values, for most species there exist a group of parameter values that have similar minimization functions (Methods). For example, for *Porites*, the optimization yields 19 ‘less optimal’ solutions whose ΔpH values lie within two standard error (i.e., 0.02 pH unit) of the best-optimized values. The estimated P, C and E values for different species, including those ‘less optimal’ solutions, exhibit positive correlations with each other (Fig. S3).

With these estimated parameters, this model predicts coral calcifying fluid DIC concentrations are elevated by a factor of ~0.9 to 2.9 relative to that of the external seawater and there exist weak correlations between the extents of calcifying fluid DIC and pH elevations within each species. Both predictions agree quantitatively with independent experimental observations (Fig. 3b). In addition, the model predicts Ca^2+^ concentrations in the calcifying fluid are similar to that of seawater, with the deviations from seawater values ranging from −2.8% to 6.8% for most species (i.e., *Acropora*, *C*. *caespitosa*, *D*. *dianthus*, *Porites* and *S*. *pistillata*) except *P*. *damicornis* and *M*. *capitata* (10.4% to 13.8%). These model predicted [Ca^2+^] values also agree with independent experimental constraints from micro-sensor measurements (e.g., within 6 ± 3% deviation from seawater [Ca^2+^] values for *Galaxea fascicularis*^37^) and estimates based on boron systematics and Raman spectroscopy (e.g., −11 ± 2% and 6 ± 6% for *Acropora yongei* and *P*. *damicornis*, respectively, under ambient seawater pH conditions^53^). Together these agreements suggest this model captures the fundamental principles governing the regulation of calcifying fluid chemistry by corals.

### Controls on calcifying fluid pH elevation: coral physiological regulation vs. seawater physicochemical condition

This numerical model takes into account different factors that can influence coral calcifying fluid chemistry, and thus enables us to isolate and evaluate the effect of each factor, particularly the relative importance of physiological regulation versus seawater conditions in controlling calcifying fluid pH.

The model shows that, at given seawater conditions, pH elevation in coral calcifying fluid is most sensitive to the P/E and C/E ratios, i.e., the interplay between proton pumping, carbon influx and the exchange of calcifying fluid with external seawater, but not to the exact values of each parameter (Fig. S3). Corals with higher P/E or lower C/E ratios are predicted to elevate their calcifying fluid pH to higher extents, e.g., ~0.2 increase in ΔpH with 20% increase of P/E ratios in *Porites* and *D*. *dianthus* (Fig. 3c).

To quantify the influence of seawater conditions (e.g., pH, DIC, T) on calcifying fluid pH elevation, two sets of model simulations were conducted with the best-optimized P, C and E values for *Porites*. First, simulations were conducted at the representative growth temperature and salinity of *Porites* corals (T = 26 °C, S = 36.4) but over a range of seawater pH and DIC concentrations. These simulations produce a suite of correlations between ΔpH and 1/β_TA_, which are negative at high pH_sw_ (>7.4~7.6) but positive at low pH_sw_ (Fig. 3d). These predicted correlations agree well with the experimental observations, and the change of correlation signs reflects the fact that seawater buffering capacity reaches a local minimum around pH_sw_ of 7.4~7.6 (Fig. S4). Moreover, the model predicted ΔpH~1/β_TA_ correlations at different seawater pH and DIC concentrations overlap over the high pH_sw_ range. This further supports that seawater buffering capacity (e.g., 1/β_TA_) exerts a fundamental control on the extents of pH elevation in coral calcifying fluid (Equation 3). In addition, these simulations predict negative correlations between [H^+^]_cf_ and seawater [DIC]/[H^+^] ratio, [TA]/[H^+^] ratio and [CO_3_^2−^], which is consistent with the observations from recent laboratory experiments (Fig. S5)^33,35^. To evaluate the effect of seawater temperature on coral calcifying pH elevation, a second set of model simulations were conducted at 20 °C (as opposed to 26 °C, the average *Porites* growth temperature, Fig. 3d). These simulations predict higher elevations of calcifying fluid pH than the simulations conducted at 26 °C, reflecting mainly the slower kinetics of aragonite precipitation at lower temperatures and thus the smaller drawdown of calcifying fluid pH by the precipitation process (Fig. 2). For example, the model predicts 33% decrease in aragonite precipitate rates in the calcifying fluid at 20 °C relative to 26 °C, at pH_sw_ = 8 and DIC_sw_ = 2200 μmol·kg^−1^.

**Figure 4:**
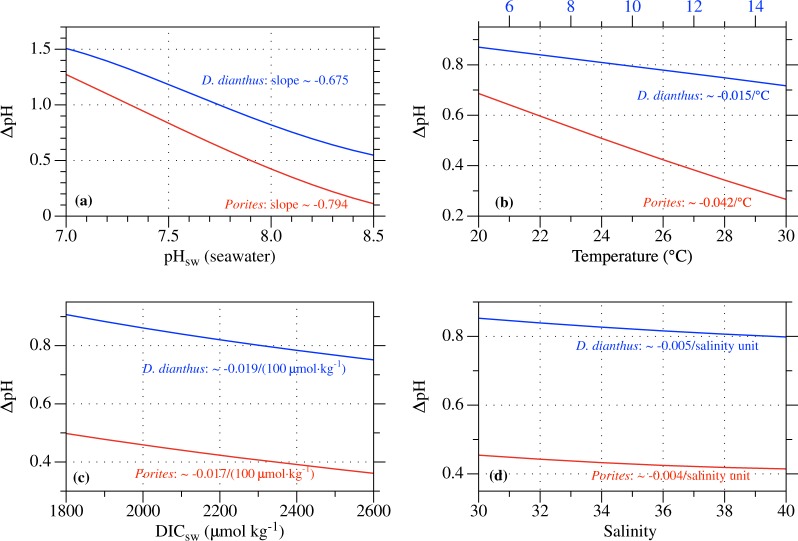
Model predicted effects of different seawater physicochemical parameters on the pH elevation in coral calcifying fluid, including seawater (**a**) pH, (**b**) temperature, (**c**) DIC concentration and (**d**) salinity. *Porites* and *D*. *dianthus* are selected to represent corals growing at warm and cold temperatures, respectively. Unless noted otherwise, model simulations were conducted with the best-optimized model parameters of each species, at pH = 8, DIC = 2200 μmol·kg^−1^ and the average growth temperature and salinity of each species, i.e., T = 26 °C and S = 36.4 (for *Porites*) and T = 8.3 °C and S = 35.2 (for *D*. *dianthus*).

More specifically, this model predicts that seawater pH exerts the strongest control on ΔpH, yielding apparent ΔpH~pH_sw_ slopes of about −0.675~−0.794 over the pH_sw_ range of 7~8.5 for different species (Fig. 4). Besides pH_sw_, seawater temperature is predicted to affect ΔpH with a sensitivity of −0.015~−0.042/ °C. In contrast, changes in seawater DIC and salinity are predicted to have much smaller effects on ΔpH, yielding sensitivities of −0.017~−0.019/(100 μmol·kg^−1^) and −0.004~−0.005, respectively. The model predicted effects of seawater pH, DIC and salinity are consistent with the respective influence of each factor on seawater buffering capacity over the studied pH_sw_ range, i.e., higher seawater pH, DIC concentration and salinity yielding higher buffering capacity and thus smaller ΔpH (Fig. S4).

To further partition the relative contributions of physiological regulation (e.g., P/E, C/E) and seawater conditions (e.g., pH, DIC, T) to the variations in ΔpH~pH_sw_ correlations observed among different coral species, model simulations were conducted for all species at their respective seawater conditions but with the same set of P, C and E parameters (e.g., the best-optimized parameter values for *Porites*). The ΔpH~pH_sw_ correlations predicted from these simulations reproduce the intra-species variations observed in each species and show even larger inter-species differences (Fig. S6). This suggests the physicochemical condition of the seawater in which these corals grew, not their physiological regulation, is the main cause of the intra- and inter-species variations observed in the coral calcifying fluid pH elevation. Experimental observations support this interpretation. For example, the offset of ΔpH~pH_sw_ correlations observed between warm-water and cold-water corals (e.g., *Porites* vs. *D*. *dianthus*, Fig. 1) is consistent with the model prediction that lower temperatures lead to slower rates of aragonite precipitation in the calcifying fluid and thus higher pH elevations. Similarly, variations in seawater temperature and DIC concentration explain the intra-species variations of ΔpH~pH_sw_ correlation observed within *D*. *dianthus*, where specimens that grew in seawaters of higher temperature and DIC concentration typically show smaller pH elevations (Fig. S7a). The model predicted DIC effect on ΔpH is also partially supported by recent long-duration laboratory manipulation experiments (8–13 weeks), where *P*. *damicornis* and *A*. *yongei* corals cultured in seawaters of the same pH and temperatures show lower pH elevations when seawater DIC_sw_ concentrations increase from ~2100 to ~3000 μmol·kg^−1^ ^30^ (Fig. S7b).

Note, however, in a shorter-duration manipulation experiment (2 weeks), *S*. *pistillata* corals show lower pH elevations at lower seawater DIC concentrations^33^ (Fig. S7c). This observation is opposite to the model predicted DIC effect and the observations for *P*. *damicornis* and *A*. *yongei* corals in longer duration experiments^30^, and indicates large DIC manipulations in these shorter experiments (i.e., from ~800 to ~2900 μmol·kg^−1^) introduced significant changes in coral physiological regulation (e.g., lower P/E and/or higher C/E ratios under lower DIC_sw_ conditions). This is consistent with the overall decrease in net photosynthesis rate and increase in respiration rate observed in the low DIC_sw_ treatments in these experiments. Similar variations in coral physiological regulation can also occur on the colony and individual scales for certain species, as different corals growing in the same seawater and different parts of the same coral can show significant variations in their calcifying fluid pH (e.g., for S. *pistillata*^14^). But the fact that variations of ΔpH~pH_sw_ correlation observed within each coral species can be reproduced by a single set of P, C and E parameters suggests physiological regulation is relatively constant within each species (Fig. 3).

### Projecting the impact of ocean acidification and warming on *Porites* calcifying fluid pH

**Figure 5:**
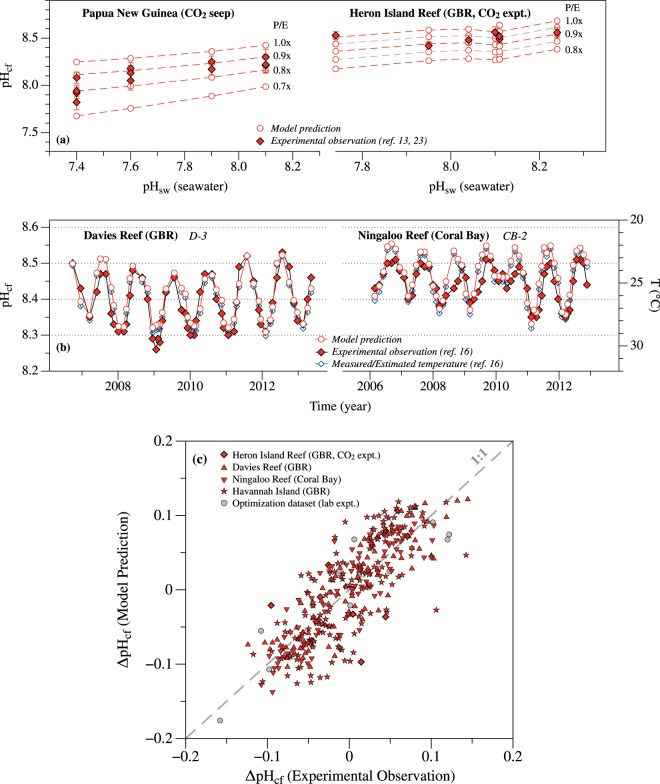
(**a**) Variability of physiological regulation (e.g., P/E ratios) in natural *Porites* colonies, determined by comparing the model predicted pH_cf_ with the experimentally measured values^13,23^. To be more consistent with the existing constraints on *Porites* calcifying fluid chemistry, the standard (i.e., ‘1.0x’) model parameter values (i.e., P, C, E) in these simulations were selected to be the optimized values that yield the closest agreement with the experimentally estimated DIC_cf_ (Fig. 3b). (**b**) Comparison of the model predicted *Porites* calcifying fluid pH with the experimentally measured values for two representative *Porites* records, along with the seasonal variations of temperature at each site^16^. Other seawater physicochemical parameters, e.g., pH and DIC, at these two sites are relatively constant at these reef sites with seasonal variations smaller than 0.08 unit and 30 μmol·kg^−1^ respectively^16^, which have negligible contributions (about 0.016 and 0.005 unit, respectively, Fig. 4) to the variations observed in *Porites* pH_cf_. Note, the temperature axis is reversed. Similar comparisons for six other natural *Porites* records^12,16^ are shown in Fig. S8. (**c**) Comparison of the model predicted changes in pH_cf_ (i.e., ΔpH_cf_, defined as the deviation of pH_cf_ from the average pH_cf_ values of each record) with the experimentally measured values. The model simulations in (**b**,**c**) were conducted with 0.95 × P/E, which are shown to represent the typical physiological regulation in *Porites* colonies around the Great Barrier Reef (e.g., Heron Island Reef, (**a**)).

The finding that seawater physicochemical condition exerts the first-order control on the pH elevation in coral calcifying fluid highlights the susceptibility of coral calcification to changes in seawater conditions. However, accurate projection of the impact of seawater changes also requires quantitative knowledge about the potential changes in coral physiological regulation in natural environments. Such changes have been implied as one of the main mechanisms for coral acclimation to ocean acidification, e.g., the apparent ‘pH homeostasis’ observed in *Porites* corals during *in situ* mesocosm experiments and at CO_2_ seep sites^13,23^. Model simulations were conducted at the same seawater conditions as reported in these mesocosm experiments and at CO_2_ seep sites, but with a range of P/E ratios (Fig. 5a). Comparison between the model predicted pH_cf_ and the experimentally measured values suggest P/E values in these natural *Porites* corals remained relatively constant during the moderate decrease of seawater pH (e.g., down to pH_sw_ of 7.6), yielding average P/E values of 90–95% of the model optimized values (Fig. 5a). Significant changes in *Porites* P/E ratios occur only with a further decrease in pH_sw_, e.g., to 80% of the model optimized values at pH_sw_ of 7.4 at CO_2_ seep sites and to 100% of the optimized values at pH_sw_ of ~7.7 in the mesocosm experiments.

Seasonal variations of pH_cf_ observed in natural *Porites* colonies also support that *Porites* corals maintain relatively constant physiological regulation. Model simulations with constant P/E ratios quantitatively reproduce the seasonal pH_cf_ variations in 8 *Porites* coral records from 3 tropical reef sites (Davies Reef, Ningaloo Reef, and Havannah Island, Figs 5 and S8)^12,16^. The exact values of P/E used in these model simulations affect the absolute values of the model predicted pH_cf_, but not the agreements between the model and experiments on the relative changes in pH_cf_ (i.e., ΔpH_cf_, the deviation of pH_cf_ from the average values of each record, Fig. 5c, RMSE = 0.039, r^2^ = 0.657, P = 2.8 × 10^−89^). These results thus suggest changes in seawater condition (more specifically, seawater temperature, Fig. 5b), as opposed to changes in coral physiology^12,16,17,28^, are responsible for most of the seasonal variations of pH_cf_ observed in these and other corals. Note, however, the model predicted [DIC]_cf_ for these corals are lower than the experimental estimates^16^ (on average a factor of 1.8 vs. 2.6 elevation relative to [DIC]_sw_, Fig. S9). Furthermore, the model predicts positive correlations between the extents of pH and DIC elevations (i.e., between DIC_cf_/DIC_sw_ and ΔpH), which are opposite to the negative correlations suggested by the geochemical data (Fig. S9). The exact cause of these discrepancies is unclear but can be related to the uncertainties associated with the geochemical method used to estimate [DIC]_cf_. Experimental DIC_cf_ estimates are derived mostly from the B/Ca composition of coral skeletons and depend strongly on the B partition coefficients used in these estimations and our understanding of the mechanism of B partition into coral skeletons^27,31,49,54–56^. Interestingly, the model predicted [DIC]_cf_ and its positive correlation with pH_cf_ agree with the geochemical constraints on the laboratory cultured *Porites* and *Pocillopora* corals when a different set of B partition coefficients are employed^27,31^.

**Figure 6:**
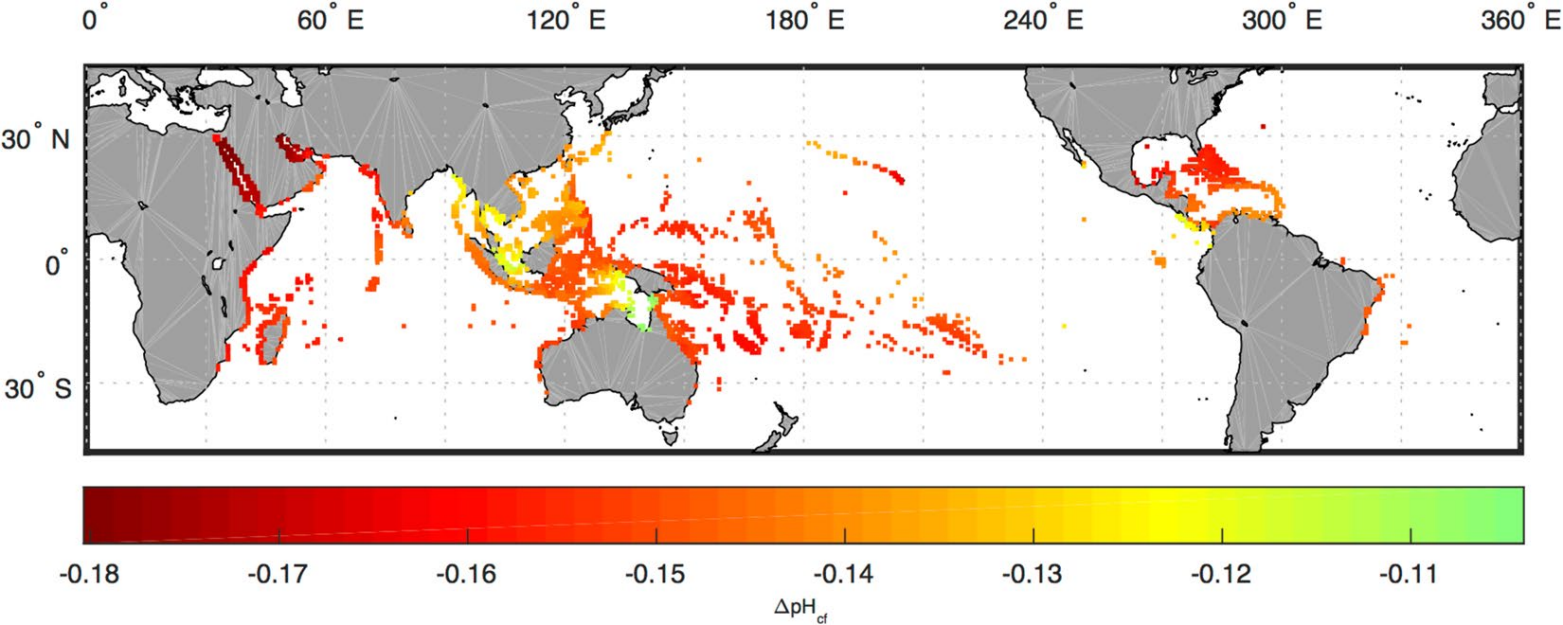
Model predicted decline in *Porites* calcifying fluid pH by the end of the 21st century due to changes in seawater physicochemical conditions. On average, the model predicts a 0.155 ± 0.024 (2σ) unit decline in *Porites* calcifying fluid pH across the global reef sites, with the largest decrease in the Red Sea and the Persian Gulf (~0.18 unit) and the smallest around the Gulf of Carpentaria (~0.11 unit). Simulations were conducted based on the outputs from the CESM-BGC RCP 8.5 run for the years 2006–2015 and 2090–2099, using the same model parameter values (i.e., P, C, E) as in Fig. 5b,c.

The fact that *Porites* corals maintain relatively constant physiological regulation on their calcifying fluid pH and the quantitative agreement between the model predicted ΔpH_cf_ and independent experimental observations in natural *Porites* colonies make it feasible to quantitatively evaluate the responses of *Porites* calcifying fluid pH to the 21 st century change in seawater conditions. For that, I force the model with outputs from the Community Earth System Model Biogeochemical run (CESM-BGC) in the RCP 8.5 projection, i.e., the ‘business as usual’ emission scenario. These simulations predict an average 0.155 ± 0.024 (2σ) unit decline in *Porites* calcifying fluid pH across the global reef sites by the end of the 21st century, with the largest decrease (~0.18 unit) predicted in the Red Sea and the Persian Gulf and the smallest around the Gulf of Carpentaria (~0.11 unit) (Fig. 6). These declines in pH_cf_ result from the combined effect of changes in each seawater physicochemical parameter, especially seawater pH and T (Fig. S10). Specifically, the decrease in seawater pH (~0.25 to 0.35 units) alone is predicted to cause an average decline of 0.076 ± 0.012 unit in *Porites* calcifying fluid pH, while the increase in temperature (~1.7 to 3 °C) is predicted to contribute to another 0.065 ± 0.012 unit decrease. In comparison, the increase in seawater DIC (~−50 to 250 µmol/kg) is expected to introduce only 0.014 ± 0.008 unit decrease in *Porites* calcifying fluid pH.

## Discussion

Up-regulation of the calcifying fluid pH and DIC is a key mechanism scleractinian corals use to promote the calcification of their carbonate skeletons. In contrast to the perception that coral physiology dominates this up-regulation process, my results show the physicochemical condition of the seawater in which corals grow, specifically the seawater temperature and buffering capacity, exerts the first-order control on the extent of calcifying fluid pH elevation and explains most of the intra- and inter-species variations. This finding, supported by both the model simulations and the existing experimental observations, highlights the susceptibility of coral calcification to changes in seawater conditions.

In particular, the influence of seawater temperature on pH elevations explains the systematic offsets between the ΔpH~pH_sw_ correlations in warm-water and cold-water corals, and reconciles the apparent discrepancy in coral ΔpH~pH_sw_ relationships observed between laboratory manipulation experiments (conducted at constant temperatures^11,14,18,19,21,22,24^) and natural reef-building corals (exposed to seasonal temperature variations^12,16,17,28^) (Fig. 1). This temperature effect arises from the temperature dependences of two components in the system, aragonite precipitation kinetics and seawater buffering capacity, with the former dominating under the typical conditions. For example, at Ω = 10, aragonite precipitation rate increases by ~120% when temperature increases from 20 °C to 25 °C^4,38^ (Equation 4), which reduces the TA and DIC concentration in coral calcifying fluid and thus lowers the calcifying fluid pH (Fig. 2). At the same time, rising temperature typically increases the seawater buffering capacity (e.g., ~13% increase in −β_TA_ from 20 °C to 25 °C, at pH = 8 and DIC = 2200 μmol·kg^−1^, Fig. S4), resulting in smaller pH elevations when the same TA and DIC changes are applied (Equation 1). Note, pH elevations in warm-water corals are predicted to have systematically stronger temperature dependences than those in cold-water corals (e.g., 0.042/ °C for *Porites* vs. 0.015/ °C for *D*. *dianthus*, Fig. 4). This reflects the larger absolute changes in aragonite precipitation rates and thus in TA and DIC fluxes at higher temperatures, for the same magnitude of temperature change. For example, at Ω = 10, aragonite precipitation rate increases by ~732 μmol·m^−2^·h^−1^when temperature increases from 20 °C to 25 °C, but it increases by only ~187 μmol·m^−2^·h^−1^ from 10 °C to 15 °C^4,38^. In comparison, other seawater physicochemical parameters (e.g., pH, DIC) affect the pH elevation in coral calcifying fluid through their influences on the seawater buffering capacity (Fig. S4, Equation 1), leading to the negative correlations between ΔpH and pH_sw_ observed in laboratory manipulation experiments and some natural corals (Fig. 1).

The finding that scleractinian corals maintain relatively constant physiological regulation of their calcifying fluid chemistry within each species and with time indicates the overall limited potential of coral acclimation to ocean acidification, and makes it feasible to quantitatively project the responses of coral calcifying fluid pH to the 21st century climate change (e.g., for *Porites*). When coupled with detailed coral skeletal growth models, these estimations shall enable more accurate predictions of coral calcification response to ocean acidification and ocean warming^57^. Note, however, this finding does not preclude differences in physiological regulation among different coral species or small variations of physiological regulation across different colonies of the same species or over time. Such inter-species differences are evident in the model optimization results, e.g., P/E and C/E ratios (Fig. S3). Furthermore, although temperature changes account for most of the seasonal pH_cf_ variations observed in tropical *Porites* corals, model simulations with constant P/E and C/E ratios overestimate the pH_cf_ during winter or underestimate the pH_cf_ during summer in some corals, e.g., by up to −0.06 unit (underestimation) and 0.04 unit (overestimation) for coral CB-2 from the Ningaloo Reef^16^ (Figs 4 and S9). These mismatches likely indicate lower P/E and/or higher C/E ratios in these *Porites* corals during winter, which could be related to the reduced respiration rate and thus less energy for enzymatic proton pumping during winter. In addition, the *Porites* coral specimen from Havannah Island shows systematically higher pH_cf_ along two of the four sampling paths and thus suggests higher coral P/E and/or lower C/E ratios during the calcification of these parts of the skeleton^12^ (Fig. S8). The comparison of model predicted pH_cf_ with experimentally measured pH_cf_ thus provides a novel way to quantify the changes of P/E and C/E ratios in corals (e.g., Fig. 5a,b) and will enable more accurate assessment of the potential of coral acclimation to OA and other environmental changes.

More generally, the results of this study validate our current understanding of coral calcification mechanism. The fact coral calcifying fluid pH can be quantitatively simulated with a seawater-based physicochemical model supports that seawater is the primary source of coral calcifying fluid and the use of coral-based geochemical proxies to reconstruct the physicochemical conditions of paleo-seawater. Moreover, by directly linking coral calcifying fluid chemistry with external seawater physicochemical parameters, this model provides a quantitative framework for better interpreting the coral-based geochemical proxies, especially the δ^11^B-pH_sw_ proxy. Existing reconstructions of seawater pH based on the boron isotope (δ^11^B) composition of coral skeletons rely on the ΔpH~pH_sw_ correlations observed in laboratory manipulation experiments conducted at constant temperatures (e.g., for *Porites*^19,21,24^) or a selection of natural specimens (e.g., for *D*. *diathus*^15,20,25^), and do not take into account other factors that can also affect coral calcifying fluid pH (e.g., T, DIC_sw_, P/E, C/E). This is problematic especially when applying this δ^11^B-pH_sw_ proxy to corals that are known to grow in environments experiencing significant changes in temperature and DIC_sw_, and would lead to incorrect pH_sw_ estimations. For example, the neglect of temperature effects on ΔpH would lead to systematic underestimation of pH_sw_ during the warmer periods and overestimation of pH_sw_ during the colder periods, and could potentially explain part of the negative correlations between seawater temperature and the reconstructed pH_sw_ in recent paleo-reconstructions^58^ and the inconsistency between these reconstructed pH_sw_ and other independent constraints^58–64^. This model makes it possible to disentangle the contributions of different factors to the variations in coral δ^11^B records and thus to derive more accurate estimates of seawater pH.

Besides scleractinian corals, many other marine calcifiers, such as calcareous foraminifera and coralline algae, also promote their calcification by up-regulating the calcifying fluid pH^65–68^. Assuming seawater is also the primary source of their calcifying fluids^65,69^, the same physicochemical principles that control the pH elevations in aqueous solutions (e.g., Equation 1) should apply to these calcifiers as well. However, the exact effects of each seawater physicochemical factor (e.g., T, pH, DIC) on the calcifying fluid pH elevation in these calcifiers depend on the specific processes involved in the pH up-regulation, and are likely to differ from those in scleractinian corals especially given that many of these calcifiers precipitate calcite not aragonite. Better understanding of the foraminifera and coralline algae calcification mechanisms in the future will enable more accurate evaluations of these effects and thus quantitative predictions of their calcification responses to the 21st century climate change.

## Methods

### Model description

The numerical model simulates the four key processes involved in coral calcification and their effects on coral calcifying fluid chemistry (e.g., TA, DIC and Ca^2+^ concentrations) with the following differential equations^43^ (Fig. 2):

$$\frac{d{[TA]}_{cf}}{dt}=P-E\times ({[TA]}_{cf}-{[TA]}_{sw})-2\times {F}_{arag}$$

$$\frac{d{[DIC]}_{cf}}{dt}=C-E\times ({[DIC]}_{cf}-{[DIC]}_{sw})-{F}_{arag}$$

$$\frac{d{[C{a}^{2+}]}_{cf}}{dt}=\frac{1}{2}\times P-E\times ({[C{a}^{2+}]}_{cf}-{[C{a}^{2+}]}_{sw})-{F}_{arag}$$

where P, C, E and F_arag_ denote the respective fluxes (per unit area) of the four key processes: (1) enzymatic pumping of proton and Ca^2+^ by Ca^2+^-ATPase, (2) CO_2_ diffusion across the cell membrane, (3) exchange of the calcifying fluid with external seawater, and (4) aragonite precipitation from the calcifying fluid. F_arag_ (per unit area) equals the rate of aragonite precipitation, R_arag_, and is calculated based on the aragonite saturation state in the calcifying fluid (Ω_cf_ = [Ca^2+^]_cf_ × [CO_3_^2−^]_cf_/K_sp_), using the temperature-dependent rate constants and reaction orders determined in laboratory carbonate precipitation experiments^4,38^ (Equation 4), where K_sp_ is the solubility product of aragonite in seawater^70^.

The steady-state solution of these equations is assumed to be representative of the final composition of the calcifying fluid and is used to calculate other carbonate parameters of the fluid (e.g., pH_cf_, [CO_3_^2−^]_cf_). During these model simulations, the dimension (length scale) of the calcifying space, defined as the volume divided by the area, is assumed to be 3 μm^44^. Experimental constraints on the dimension of the calcifying space are currently limited^71–75^, and the existing estimates range from 0.3 to 30 μm^44,75^. Note, however, the dimension of the calcifying space assumed in the model affects only the time for the system to reach the steady state but not the composition at the steady state^44^. It thus has negligible effects on the model optimization results and the model predicted calcifying fluid pH.

Besides the steady-state model as employed in this study, closed-system batch models have also been proposed to simulate coral calcification^76,77^. In these models, parcels of seawater are transported to the calcifying space, processed and then expelled. Existing geochemical constraints cannot distinguish between these two types of models^47^. A steady-state model is adopted here because it involves fewer assumptions and to some degree encompasses the batch models (e.g., when E = 0).

### Optimization of model parameters

Values of model parameters P, C and E were assumed to be the same for all coral individuals within the same species, and were optimized to reproduce the observed ΔpH~pH_sw_ relationship for each species, specifically by minimizing the following least squares function:

$$Min=\sum _{i}{(\frac{p{H}_{model,i}-p{H}_{meas,i}}{SF})}^{2}$$

where subscripts ‘model’ and ‘meas’ refer to the modeled and measured values of coral calcifying fluid pH, and SF represents a scaling factor and was set to be the measured pH values. To ensure the robustness of the optimization results, only coral species whose pH_sw_ variations cover a sufficient range (≥0.3 units) are selected for parameter optimization. These include *Acropora*, *C*. *caespitosa*, *D*. *dianthus*, *M*. *capitata*, *Porites*, *P*. *damicornis* and *S*. *pistillata*.

During the optimization, the measured seawater conditions associated with each coral sample were adopted as the model inputs. Both P and C values were allowed to vary from 0 to 30.7 μmol·m^−2^·s^−1^, corresponding to 0 to 1 × 10^4^ μmol·s^−1^ for every kilogram of calcifying fluid (with the assumed dimension of the calcifying space of 3 μm). Similarly, E values were allowed to vary within the range corresponding to the calcifying fluid turnover time of 1s to 5.7 h, to be consistent with the limited experimental constraints (i.e., <2 min to <5.7 h^47,78^). Note, as discussed in the main text, the model results are not sensitive to the exact values of each parameter but to their relative ratios (i.e., P/E and C/E). The optimizations were performed using the Matlab program, with 1000 distinct starting points for each optimization.

Note, coral calcifying fluid pH has been experimentally constrained with a variety of geochemical methods, including boron isotope analysis of coral skeleton^11–31^, *in situ* measurements with pH-sensitive dyes^14,33–35^ and pH microelectrodes^6,36,37^. The optimization of model parameters in this study is based on pH_cf_ estimates derived from boron isotope analysis of coral skeleton. These data are selected because they are integrated over weeks (or longer) of coral calcification process and thus are more representative of the average pH condition of coral calcifying fluid, as compared to pH estimates from pH sensitive dyes and *in situ* microelectrodes measurements. The conclusion of this study does not depend on the pH_cf_ dataset used, as pH_cf_ estimates from these different methods corroborate each other and all show consistent negative correlations between ΔpH and pH_sw_ in laboratory manipulation experiments.

### Projection of *Porites* calcifying fluid pH responses

Projection of *Porites* calcifying fluid pH responses to the 21st century climate change was made for different reefs based on outputs from the CESM-BGC RCP 8.5 prediction run. Projections of seawater pH, DIC, T, and S were extracted from the 1° × 1° model and averaged over the first ten (2006–2015) and last ten (2090–2099) years to represent the current and end of century seawater conditions at different reef sites around the globe. Reef site locations are provided by the ReefBase database^79^. Model parameters P (proton pumping), C (carbon influx) and E (exchange of the calcifying fluid with external seawater) were prescribed at the optimized values that yield the best agreement with the experimentally estimated pH_cf_ and DIC_cf_ in natural *Porites* corals (Fig. 5), and were held constant for the predictions over the 21th century.

## Supplementary information

SuppIementary Information

## Data Availability

All data generated or analyzed during this study are included in this article (and its Supplementary Information files), and are also available from the corresponding author on reasonable request.
