# Supplementary material for: Seawater temperature and buffering capacity modulate coral calcifying pH
Source: Sci Rep. 2019 Feb 4;9:1189. doi: 10.1038/s41598-018-36817-y (PMC6362028; doi:10.1038/s41598-018-36817-y)
Supplement: Supplementary file 1 — SuppIementary Information [file 41598_2018_36817_MOESM1_ESM.pdf]

Supplementary Information for

Seawater temperature and buffering capacity modulate coral calcifying pH

Weifu Guo[*]
Department of Geology and Geophysics, Woods Hole Oceanographic Institution, Woods Hole, MA 02543

[*]To whom correspondence should be addressed. Email: wfguo@whoi.edu

Contents:

Supplementary Figures S1 to S10
Supplementary Table S1
Supplementary References

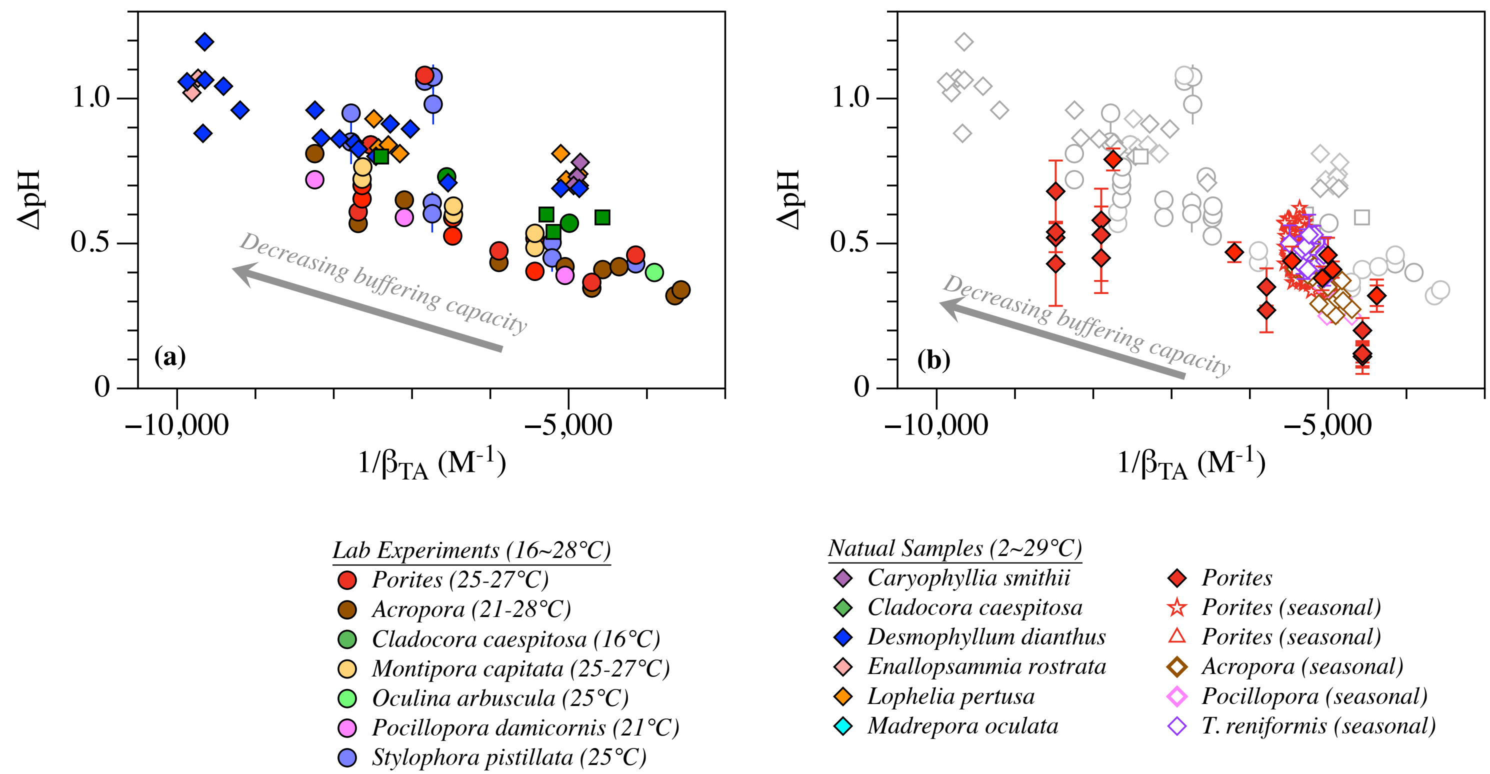

Figure S1. Negative correlations between pH elevation in coral calcifying fluid (ΔpH) and seawater buffering capacity (expressed as $1/\beta_{TA}$). These correlations are consistent with what expected from the physicochemical principles (Equation 3, see text). Regardless of the species they belong to, corals that grow in seawaters of similar temperature and buffering capacity show similar extents of pH elevation. This supports that seawater buffering capacity exerts a fundamental control on coral calcifying fluid pH elevation.

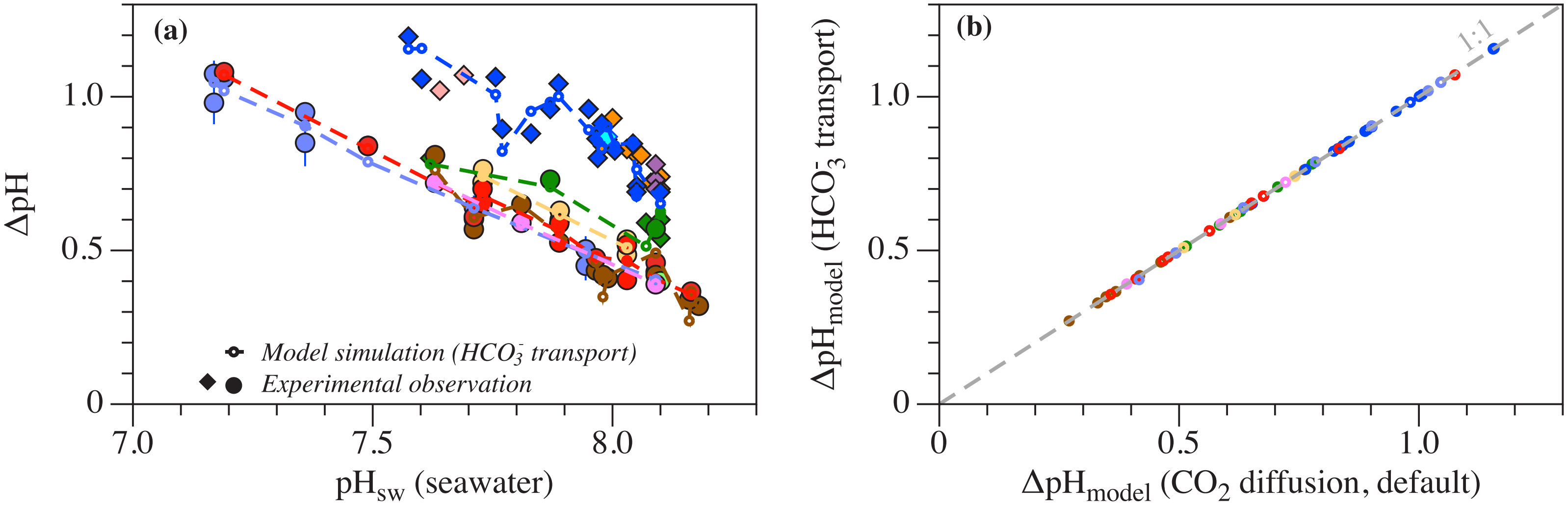

Figure S2. Model simulation of the regulation of calcifying fluid chemistry by corals, assuming HCO_3^- transport as the source of carbon influx for coral calcification (as opposed to CO_2 diffusion; Fig. 2, see text). **(a)** Comparison between the model simulated calcifying fluid pH elevation (open circles) and the experimentally measured values for each species (filled symbols). The small open circles represent the best-optimized model results and are connected by dashed lines for better illustration. **(b)** Comparison of the model simulated calcifying fluid pH elevations between two types of models, assuming HCO_3^- transport and CO_2 diffusion as the source of carbon for coral calcification, respectively. The agreement between the results from these two types of models is expected, because the effect of HCO_3^- transport on the calcifying fluid TA and DIC (increasing TA_{cf} and DIC_{cf} with a 1:1 ratio) has already been implicitly accounted for in the CO_2 diffusion model through the combination of proton pumping (increasing only TA_{cf}) and CO_2 diffusion (increasing only DIC_{cf}). Therefore, this study focuses on the results from the CO_2 diffusion model.

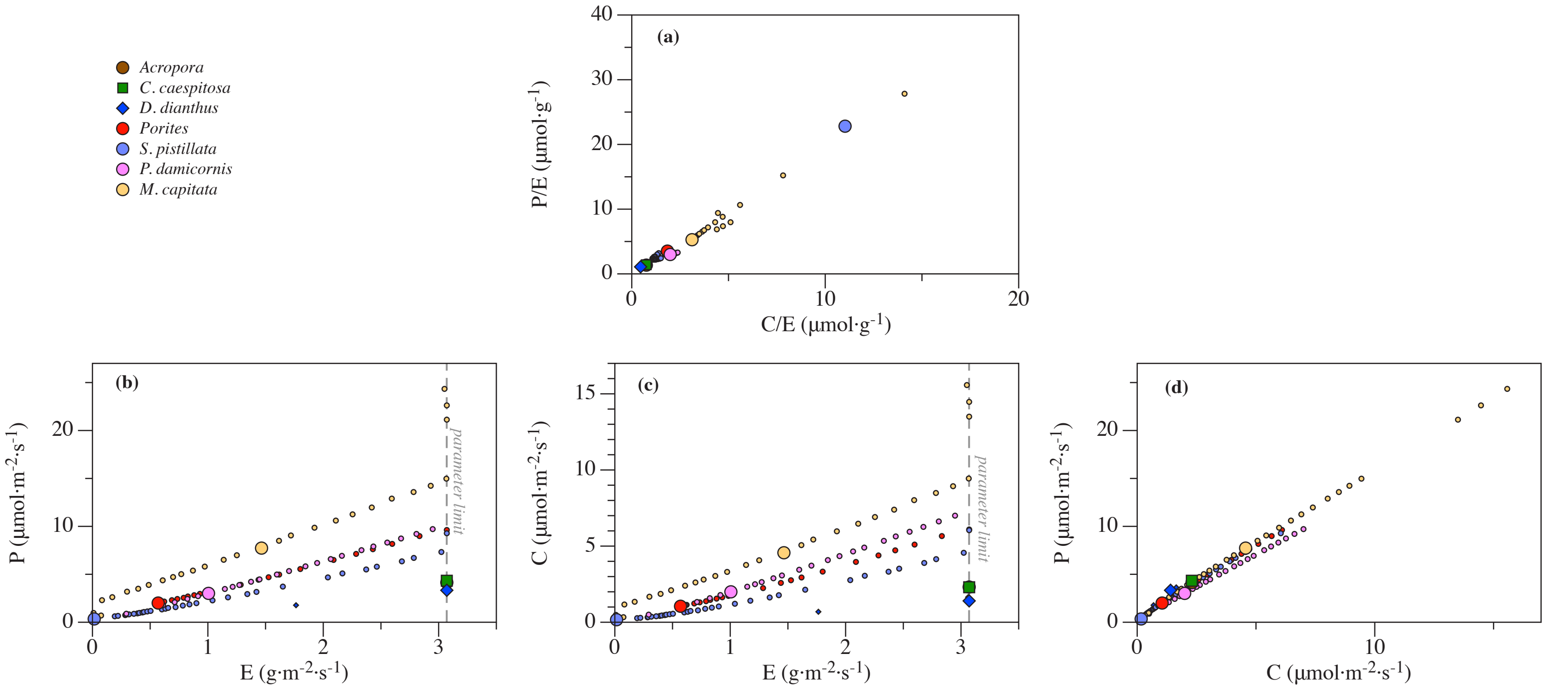

Figure S3. Correlations between the optimized values of three key model parameters, enzymatic proton pumping (P), carbon influx (C) and exchange of the calcifying fluid with external seawater (E). The large symbols denote the parameter values that best reproduce the experimentally observed $\Delta pH \sim pH_{sw}$ correlations, while the smaller symbols denote the 'less optimal' values whose predicted pH_{cf} values fall within two standard error (i.e., 0.02 pH unit) of the predictions from the best-estimated parameters. Note, the symbols for *Acropora* and *C. caespitosa* overlap in the figures because the optimized parameter values for these two species are very similar (Table S1).

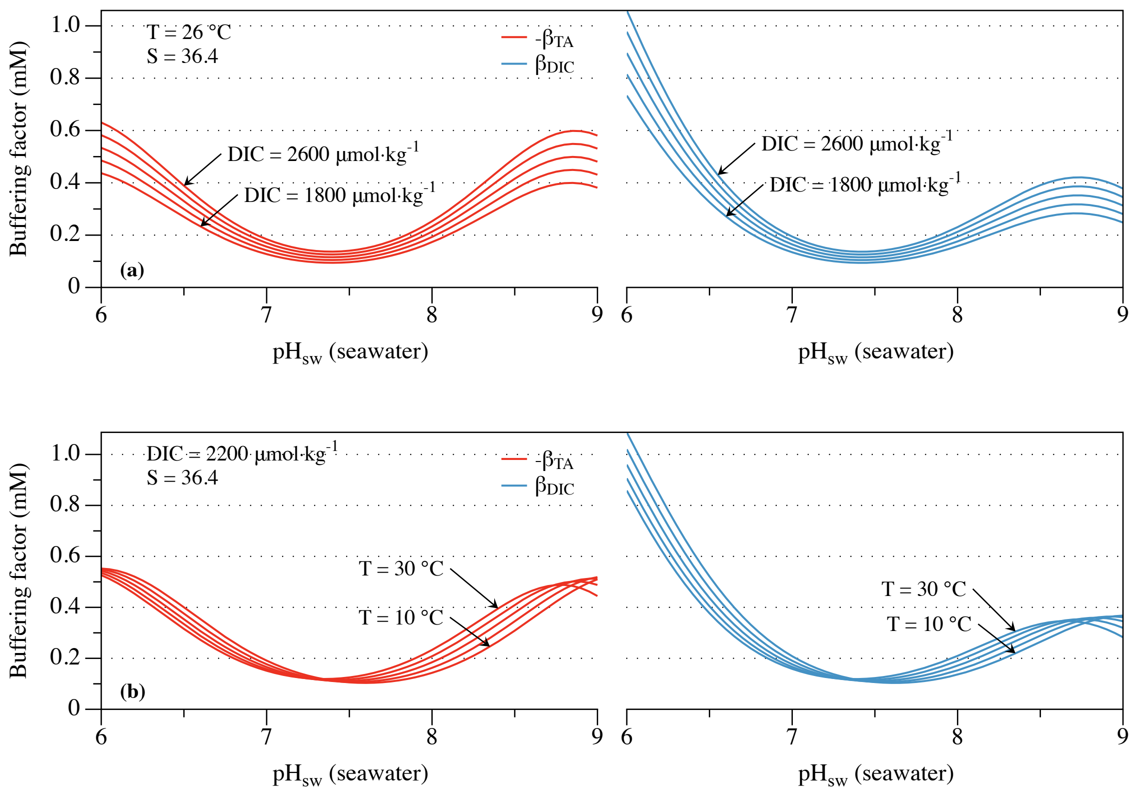

Figure S4. Dependence of seawater buffering capacity on **(a)** pH and DIC concentration and **(b)** temperature. Calculations in (a) were conducted at the average temperature and salinity of *Porites* samples (T = 26°C, S = 36.4), over a pH range of 6 to 9 and DIC concentrations of 1800 to 2600 $\mu mol \cdot kg^{-1}$. Similarly, calculations in (b) were conducted at S = 36.4 and DIC = 2200 $\mu mol \cdot kg^{-1}$, over a pH range of 6 to 9 and temperature range of 10 to 30°C. Seawater buffering capacity, expressed as the commonly defined buffering factors β_{TA} and β_{DIC}[1], reaches one of the minimums in (a) when pH is ~7.4.

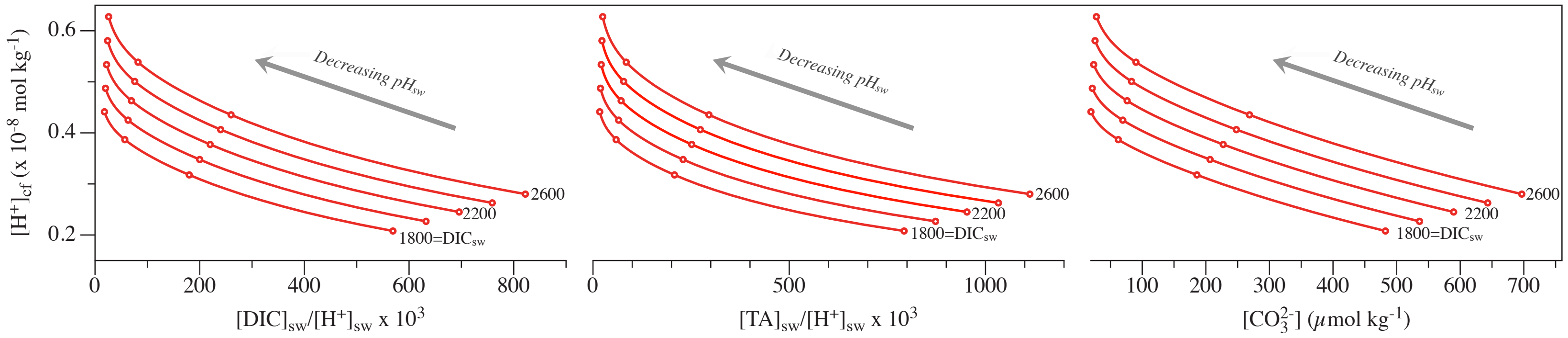

Figure S5. Model predicted negative correlations between $[H^+]_{cf}$ and seawater $[DIC]/[H^+]$ ratio, $[TA]/[H^+]$ ratio and $[CO_3^{2-}]$. These predictions are consistent with the observations from some laboratory manipulation experiments[2,3]. The model simulations were conducted in the same way as in Fig. 3d using the best-optimized parameters for *Porites*, with pH and DIC concentrations varying from 7 to 8.5 and from 1800 to 2600 $\mu mol \cdot kg^{-1}$ respectively. Open circles on the model lines represent the results when $pH_{sw} = 7, 7.5, 8$ and 8.5.

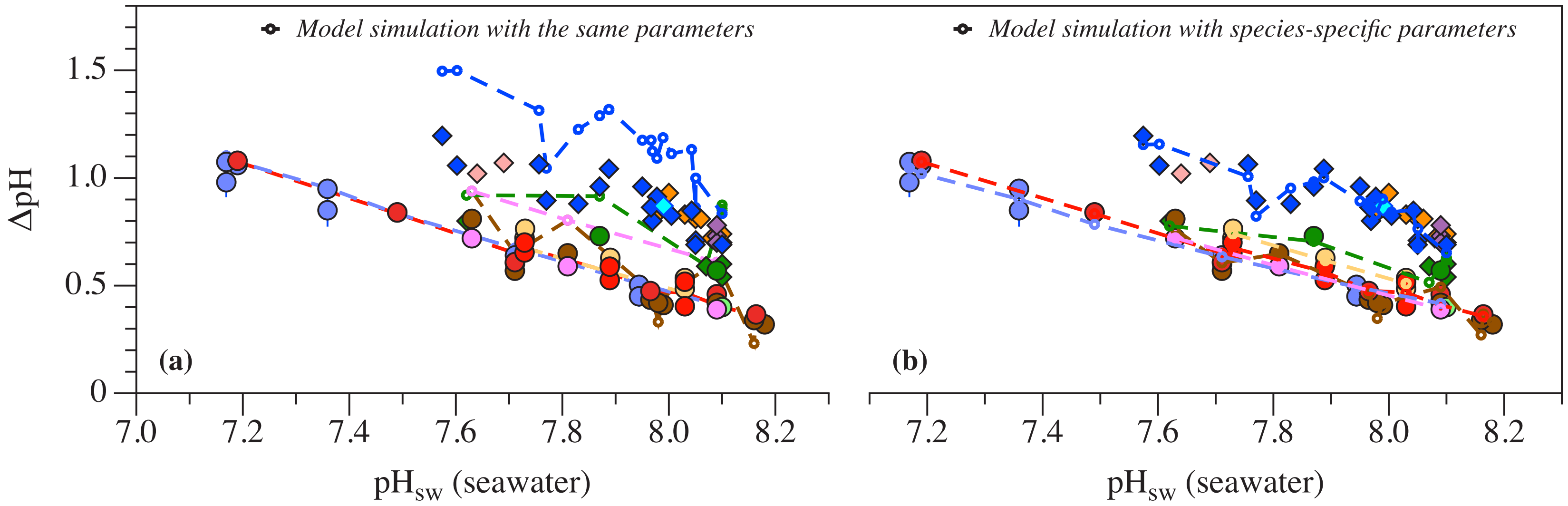

Figure S6. (a) Model predicted calcifying fluid pH elevation for different species with the same set of P, C and E values. In comparison, the model predictions with species-specific parameters (i.e., Fig. 3a) are also shown **(b)**. The fact the simulations with the same parameter values reproduce intra-species variations observed in each species but show even larger inter-species differences, suggests physicochemical conditions of the seawater in which corals grow, as opposed to the difference in physiological regulation, is the main cause of the intra- and inter-species variations observed in coral calcifying fluid pH elevation. The parameter values used in the simulations in (a) are the best-optimized parameter values for *Porites*.

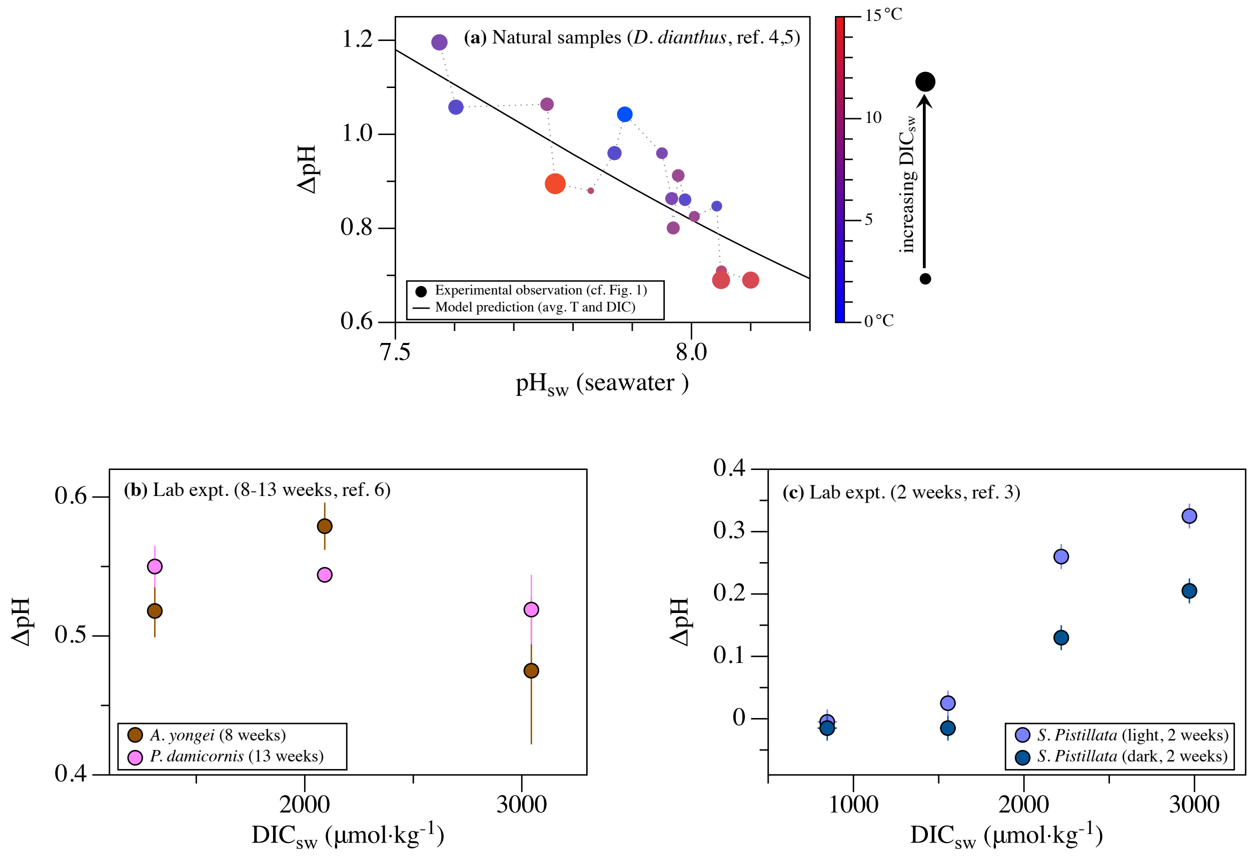

Figure S7. Experimental evidence supporting the model predicted effects of seawater T and DIC concentration on the pH elevation in coral calcifying fluid. **(a)** Variation of pH elevation in natural *D. dianthus* samples[4,5], showing smaller extents of pH elevation at higher seawater temperatures and DIC concentrations (redder and larger symbols). This is consistent with the model predicted effects for these seawater physicochemical parameters. **(b-c)** Correlation between coral calcifying fluid pH elevation and seawater DIC concentrations in laboratory manipulation experiments[3,6]: (b) *A. yongei*, *P. damicornis* and (c) *S. Pistillata* corals (see main text for details). The black line in (a) denotes the model prediction at the average seawater temperature and DIC concentration for these specimens using the best-optimized parameter values (i.e., P, C, and E) for *D. dianthus*.

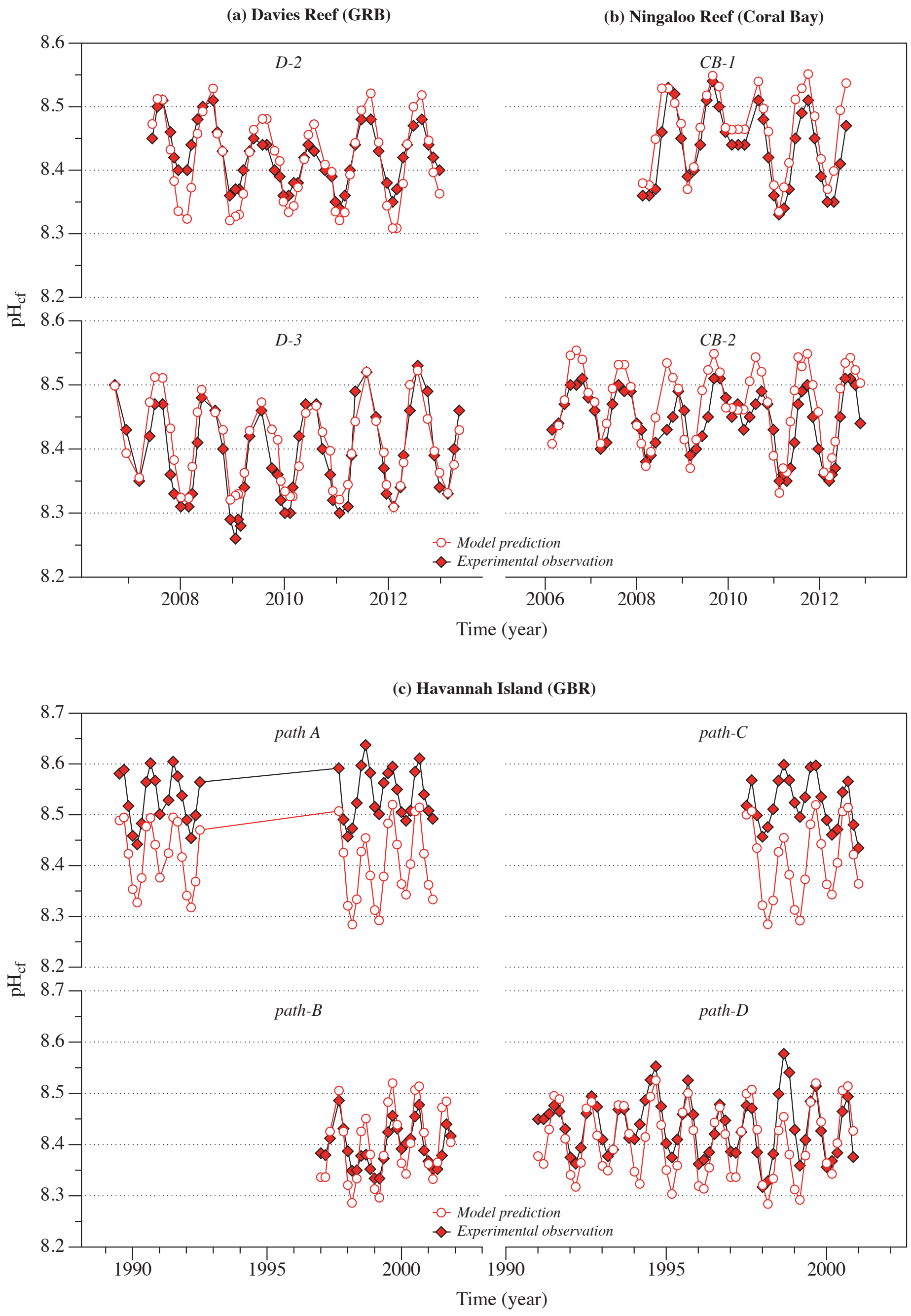

Figure S8. Comparison of the model predicted calcifying fluid pH with the experimentally measured values for 8 *Porites* core records from 3 reef sites[7,8]. The model simulations were conducted with the same model parameter (i.e., P, C, E) values as in Fig. 5b, which is shown to represent the typical physiological regulation in *Porites* colonies around the Great Barrier Reef (Fig. 5a).

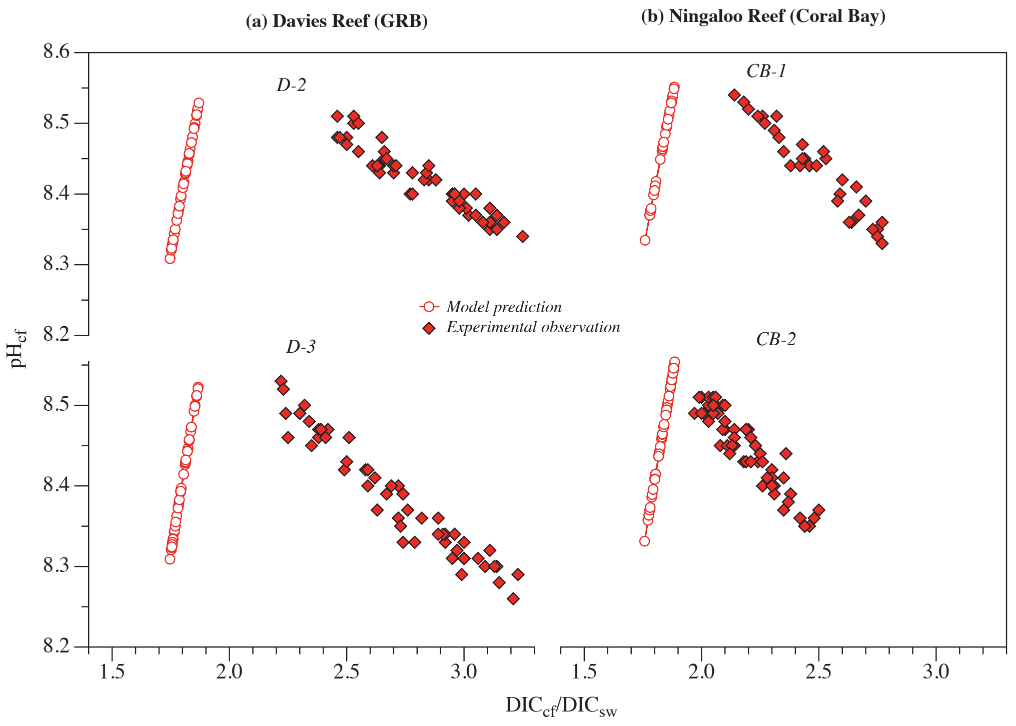

Figure S9. Model predicted positive correlations between pH elevation and DIC elevation in 4 natural *Porites* cores. This prediction is different from the negative correlation suggested by the existing experimental estimates[7]. The discrepancy is likely related to the uncertainties associated with the geochemical methods used to derive DIC_{cf} estimates, especially the boron partition coefficients used in these estimations (see text). The model simulations were conducted with the same model parameter (i.e., P, C, E) values as in Fig. 5b and S8.

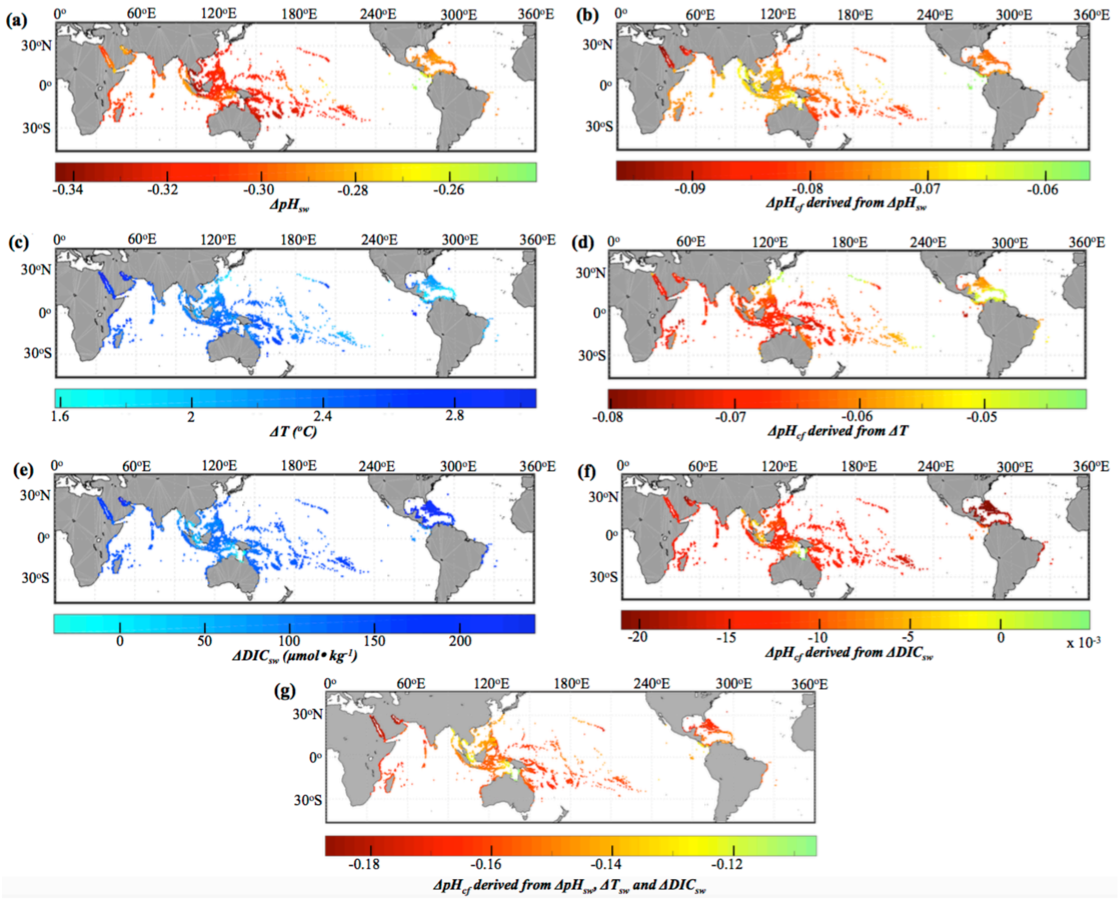

Figure S10. Projected changes in seawater (a) pH, (c) T and (e) DIC and the corresponding changes in *Porites* calcifying fluid pH (b, d, f respectively) by the end of the 21st century. The projected change in *Porites* calcifying fluid pH derived from the combined effects is shown in the bottom panel (g). Simulations were conducted based on the outputs from the CESM-BGC RCP 8.5 run for the years 2006-2015 and 2090-2099, using the same model parameter (i.e., P, C, E) values as in Fig. 5b.

Table S1. Best optimized model parameter values (P, C, and E) for different coral species.

Coral Species	Best Optimized Value		
	**P** ($\mu mol \cdot m^{-2} \cdot s^{-1}$)	**C** ($\mu mol \cdot m^{-2} \cdot s^{-1}$)	**E** ($g \cdot m^{-2} \cdot s^{-1}$)
Acropora	4.15	2.31	3.07
C. caespitosa	4.33	2.30	3.07
D. dianthus	3.33	1.40	3.07
Porites	2.00	1.05	0.57
S. pistillata	0.35	0.17	0.015
P. damicornis	3.01	2.00	1.01
M. capitata	7.73	4.56	1.47

Supplementary References

1 Egleston, E. S., Sabine, C. L. & Morel, F. M. M. Revelle revisited: Buffer factors that quantify the response of ocean chemistry to changes in DIC and alkalinity. *Global Biogeochem. Cycles* **24**, GB1002 (2010).

2 Venn, A. A. *et al.* Impact of seawater acidification on pH at the tissue-skeleton interface and calcification in reef corals. *Proc. Nat. Acad. Sci. U.S.A.* **110**, 1634-1639 (2013).

3 Comeau, S. *et al.* Coral calcifying fluid pH is modulated by seawater carbonate chemistry not solely seawater pH. *Proc. Roy. Soc. London, Ser. B* **284**, 20161669 (2017).

4 McCulloch, M. *et al.* Resilience of cold-water scleractinian corals to ocean acidification: Boron isotopic systematics of pH and saturation state up-regulation. *Geochim. Cosmochim. Acta* **87**, 21-34 (2012).

5 Anagnostou, E., Huang, K. F., You, C. F., Sikes, E. L. & Sherrell, R. M. Evaluation of boron isotope ratio as a pH proxy in the deep sea coral *Desmophyllum dianthus*: Evidence of physiological pH adjustment. *Earth Planet. Sci. Lett.* **349**, 251-260 (2012).

6 Comeau, S., Cornwall, C. E., DeCarlo, T. M., Krieger, E. & McCulloch, M. T. Similar controls on calcification under ocean acidification across unrelated coral reef taxa. *Glob. Change Biol.* **24**, 4857–4868 (2018).

7 McCulloch, M. T., D'Olivo, J. P., Falter, J., Holcomb, M. & Trotter, J. A. Coral calcification in a changing world and the interactive dynamics of pH and DIC upregulation. *Nature Comm.* **8**, 15686; 10.1038/ncomms15686 (2017).

8 D'Olivo, J. P. & McCulloch, M. T. Response of coral calcification and calcifying fluid composition to thermally induced bleaching stress. *Sci. Rep.* **7**, 2207; 10.1038/s41598-017-02306-x (2017).
